# Fibronectin Unfolding Revisited: Modeling Cell Traction-Mediated Unfolding of the Tenth Type-III Repeat

**DOI:** 10.1371/journal.pone.0002373

**Published:** 2008-06-11

**Authors:** Elaine P. S. Gee, Donald E. Ingber, Collin M. Stultz

**Affiliations:** 1 Graduate Program in Biophysics, Harvard University, Cambridge, Massachusetts, United States of America; 2 Department of Surgery, Vascular Biology Program, Children's Hospital, Harvard Medical School, Boston, Massachusetts, United States of America; 3 Department of Pathology, Vascular Biology Program, Children's Hospital, Harvard Medical School, Boston, Massachusetts, United States of America; 4 Department of Electrical Engineering and Computer Science, Research Laboratory of Electronics, Harvard-MIT Division of Health Sciences and Technology, Massachusetts Institute of Technology, Cambridge, Massachusetts, United States of America; Illinois Institute of Technology, United States of America

## Abstract

Fibronectin polymerization is essential for the development and repair of the extracellular matrix. Consequently, deciphering the mechanism of fibronectin fibril formation is of immense interest. Fibronectin fibrillogenesis is driven by cell-traction forces that mechanically unfold particular modules within fibronectin. Previously, mechanical unfolding of fibronectin has been modeled by applying tensile forces at the N- and C-termini of fibronectin domains; however, physiological loading is likely focused on the solvent-exposed RGD loop in the 10^th^ type-III repeat of fibronectin (10FNIII), which mediates binding to cell-surface integrin receptors. In this work we used steered molecular dynamics to study the mechanical unfolding of 10FNIII under tensile force applied at this RGD site. We demonstrate that mechanically unfolding 10FNIII by pulling at the RGD site requires less work than unfolding by pulling at the N- and C- termini. Moreover, pulling at the N- and C-termini leads to 10FNIII unfolding along several pathways while pulling on the RGD site leads to a single exclusive unfolding pathway that includes a partially unfolded intermediate with exposed hydrophobic N-terminal β-strands – residues that may facilitate fibronectin self-association. Additional mechanical unfolding triggers an essential arginine residue, which is required for high affinity binding to integrins, to move to a position far from the integrin binding site. This cell traction-induced conformational change may promote cell detachment after important partially unfolded kinetic intermediates are formed. These data suggest a novel mechanism that explains how cell-mediated forces promote fibronectin fibrillogenesis and how cell surface integrins detach from newly forming fibrils. This process enables cells to bind and unfold additional fibronectin modules – a method that propagates matrix assembly.

## Introduction

Fibronectin (FN) is an extracellular matrix (ECM) protein that plays an important role in cell adhesion, growth, and survival during embryological development and is critical for wound healing and maintenance of normal tissue architecture throughout adult life [1]–[3]. FN is secreted as a soluble dimer but forms fibrils in the extracellular space [4]. Cells bind to FN molecules through transmembrane integrin receptors that recognize a conserved solvent-exposed RGD loop within the cell-binding region of FN [5]–[7]. Binding of cell-surface integrins is necessary for FN fibrillogenesis; e.g., antibodies that prevent binding of FN to integrins inhibit the formation of FN fibrils *in vitro* [8]. However, the binding of FN to integrins is not sufficient to initiate fibril formation; instead cells must also exert cytoskeletally-generated traction forces on their cell surface integrins, which transfer the force to underlying FN adhesions to promote FN fibril assembly [9], [10]. Cells therefore trigger FN fibrillogenesis and remodel the ECM by exerting mechanical stresses at a specific position in the FN molecule – the RGD site that mediates cell-surface integrin binding [4], [6], [11].

FN is a multidomain protein where each domain can be classified into one of three distinct types (I, II, or III). The 10^th^ type III repeat (10FNIII) contains the RGD site that is required for integrin recognition. Molecular force spectroscopy studies have revealed that this module is the most compliant, and thus it has been suggested that 10FNIII unfolds in response to cell-traction forces and that this unfolding facilitates FN fibrillogenesis [12]. While various aspects of this hypothesis have been debated in the literature [13], [14], there are data to suggest that 10FNIII unfolding plays a role in FN fibril formation. For example, recent studies suggest that the force generated during actin-myosin mediated cell traction is sufficient to unfold a FNIII module and that 10FNIII unfolding may facilitate the formation of FN fibrils *in vitro* [15], [16]. Consequently, characterization of specific regions of 10FNIII that become exposed when mechanically strained may offer insight into the earliest steps of FN fibrillogenesis.

Studying the effects of mechanical strain on the structure of 10FNIII is problematic because the direct observation of short-lived, partially unfolded states is difficult to achieve experimentally. Computer simulations provide a useful approach to address this problem. Molecular dynamics simulations, for example, have shed light on the physical basis of the mechanical stability of titin – a modular protein consisting of repeating FNIII-like domains – and of FN [17]–[21]. But in all of these simulations the molecules were mechanically strained by applying tensile force to the N- and C-termini of the 10FNIII domain [17]–[21]. This loading pattern may not be helpful for understanding cell-traction induced fibrillogenesis as it is more relevant to understand how forces exerted through integrins bound to the RGD-loop of 10FNIII influence 10FNIII unfolding. Consequently, the goal of this study was to uncover molecular details of force-mediated unfolding of the 10FNIII domain under physiological loading conditions by applying tension along an axis that includes the integrin-binding RGD loop.

Our data suggest that pulling at the RGD motif leads to partial unfolding of 10FNIII along a unique and well-defined pathway. By contrast, pulling at the N- and C-termini leads to unfolding that can occur along many different pathways. Unfolding along a unique and robust pathway provides a mechanism to enable FN to reliably sample a limited number of intermediate structures that may preferentially promote FN fibril formation. These studies also reveal a novel uncoupling mechanism whereby traction-induced partial unfolding of the 10FNIII domain dislodges FN-integrin adhesions; this uncoupling permits new FN-integrin binding and propagation of fibrillogenesis.

## Results

### Two Models of Force-induced 10FNIII Unfolding

**Figure 1:**
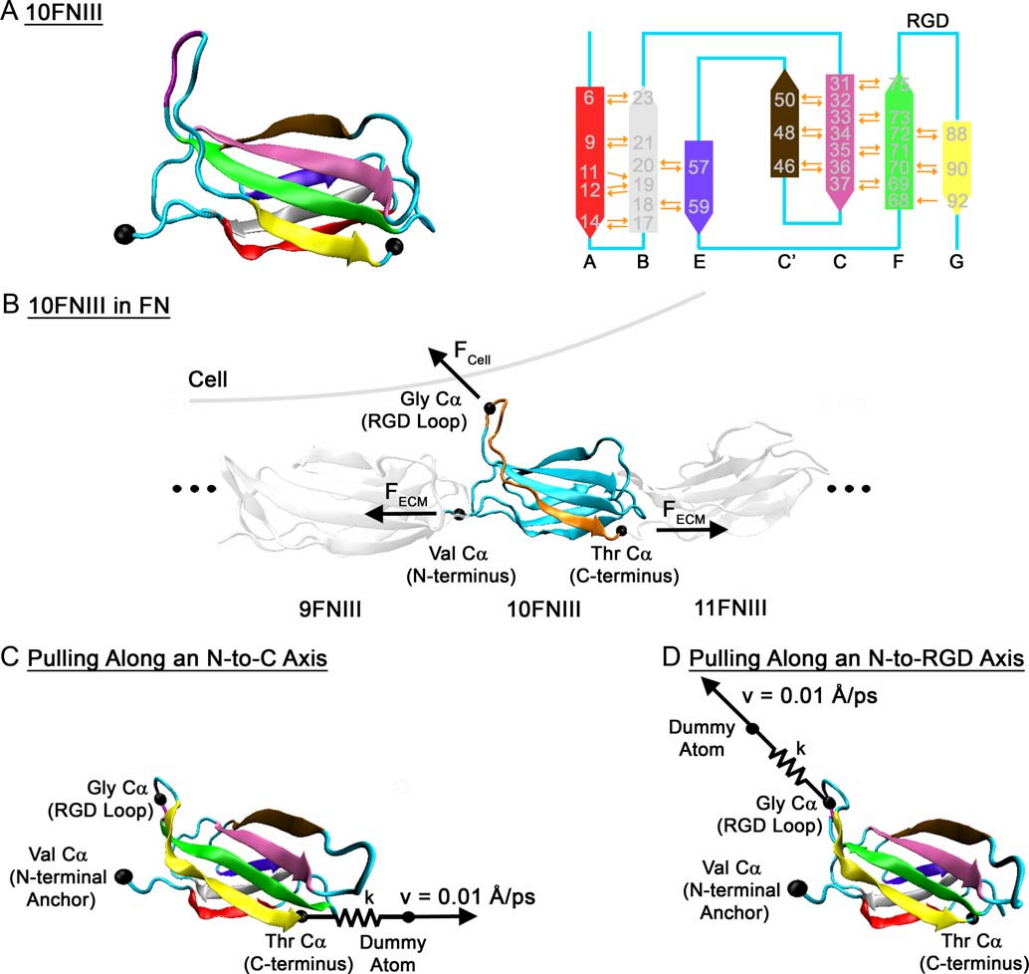
Local Environment of 10FNIII. (A) Crystal structure of 10FNIII. On the left is the cell-binding module of FN (type III), whose globular folded form is composed of seven β-strands split between two β-sandwich sheets. The RGD loop is highlighted as purple in the loop connecting β-strands *F* and *G*. N- and C- termini are labeled as black spheres. On the right is a schematic topology map of the secondary structure of 10FNIII with each β-strand labeled and color-coded corresponding to the crystal structure on the left. β-strand boundaries are adapted from the crystal structure as previously shown [17]. Residues participating in hydrogen bonds (orange arrows pointing in the direction from hydrogen bond donor to acceptor) are labeled in gray. Hydrogen bonds shown are between backbone atoms of the β-strand residues and are calculated for distances within 3.5 Å and angles within 120°–180° using the software VMD. (B) 10FNIII as it sits in FN. 10FNIII, adjacent to the ninth (9FNIII) and eleventh (11FNIII) type III modules, experiences two types of forces. The cell can directly apply force (F_CELL_) at the RGD loop by binding through a cell-surface integrin and applying cell traction. Force can also be applied to FN indirectly through the ECM (F_ECM_). F_ECM_ represents the forces generated by cells bound to remote sites in the ECM that are propagated to 10FNIII through neighboring domains bonded at the N- and C- termini. Residues C-terminal to the RGD loop (highlighted in gold) adopt a relatively extended conformation. (C) Pulling along an N-to-C axis. The protein is anchored at the Cα atom of the N-terminal residue (Val1416), and force is applied through a spring (with spring constant *k*) attached at the pulling point by translating the free end (attached to a dummy atom) at constant velocity (v = 0.01 Å/ps) in the defined direction. This process transmits force to the attached Cα atom of the C-terminal residue (Thr1509). Each β–strand is color-coded and labeled as in (A). (D) Pulling along an N-to-RGD axis. The Cα atom of the N-terminus is anchored, and the pulling force is exerted through a spring attached to the Cα atom of the Gly residue in the RGD tripeptide. Each β–strand is color-coded and labeled as in (A).

To mechanically unfold 10FNIII (Figure 1A), two points of contact are necessary – a fixed anchoring point and a pulling point where tensile forces are applied. As 10FNIII is covalently bonded to two other type-III domains at its N- and C- termini (Figure 1B), the use of these termini as anchoring and pulling points (N-to-C pulling; Figure 1C), as done in past molecular dynamics simulations [17]–[21], models structural changes in 10FNIII when external tension at distant sites is propagated to 10FNIII through neighboring modules. Because mechanical unfolding of 10FNIII likely occurs when cell-generated tensile force is applied through integrins at the RGD site (Figure 1B), we simplified the integrin-RGD interface such that the forces transferred to 10FNIII are modeled at a single pulling point - the Cα-carbon of the central glycine residue in the RGD triplet – with the fixed anchor point defined at the N-terminus (N-to-RGD pulling; Figure 1D). Alternatively, one could choose to anchor at the C-terminus, however this does not lead to module unfolding because the RGD site and the C-terminus are separated by thirteen residues that adopt a relatively extended conformation (Figure 1B) (data not shown). Moreover, the N-terminal region of full length fibronectin, which contains all FN repeats, contains binding sites for other ECM proteins such as collagen, fibrin, and other FN modules [22]. Therefore anchoring at the N-terminus of 10FNIII (Val in Figure 1B) models the scenario where the upstream FN modules are tightly bound to these components and therefore remains relatively stationary, while cellular forces are applied at the RGD loop.

### Different 10FNIII Unfolding Pathways under Different Loading Conditions

**Figure 2:**
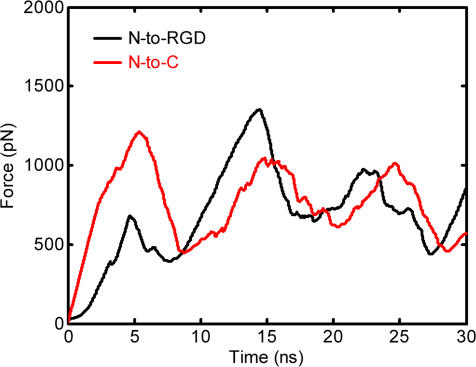
Extension Force Associated with Unfolding 10FNIII Along Either Axis. Averaging across each of the ten simulations along the N-to-RGD (black) or N-to-C (red) axis shows that the general properties of the force profiles are similar.

Constant velocity steered molecular dynamics (SMD) simulations were used to study structural changes in 10FNIII when tension is applied along both axes (N-to-C or N-to-RGD). Ten independent force-mediated unfolding simulations (30 ns each) were performed for each pulling axis. External force at the pulling point is provided by translating, at constant velocity, the free end of a spring (dummy atom in Figure 1C,D) that is attached to the pulling point. The rate at which 10FNIII unfolds is therefore a function of the velocity of the dummy atom. Fast unfolding simulations minimize the time required to unfold the protein but may introduce artifacts into the unfolding trajectories as the unfolding rate is much faster than the true unfolding rate *in vivo*; slower simulations are more realistic, but they require significant computational resources. Therefore, the optimal choice of spring extension represents a compromise between two competing considerations – computational efficiency and accuracy. Prior studies demonstrate that meaningful insights into the unfolding mechanism of fibronectin modules can be obtained from simulations performed at pulling rates much faster than *in vivo* unfolding rates [18], [20]. In the present case, an extension velocity of v = 0.01 Å/ps was used; this rate is at least fifty times slower than that used in past simulations of force-mediated unfolding of 10FNIII [18], [20]. These data reveal that the overall shape of the unfolding force profiles is similar for the N-to-C and N-to-RGD unfolding trajectories, with both exhibiting major peaks of similar number and timing (Figure 2). There exist differences in the relative magnitudes of the force barriers, especially during the early stages of partial unfolding when relatively small forces were required to mechanically unfold 10FNIII along the N-to-RGD axis (e.g., time<10 ns) compared to the N-to-C axis.

**Figure 3:**
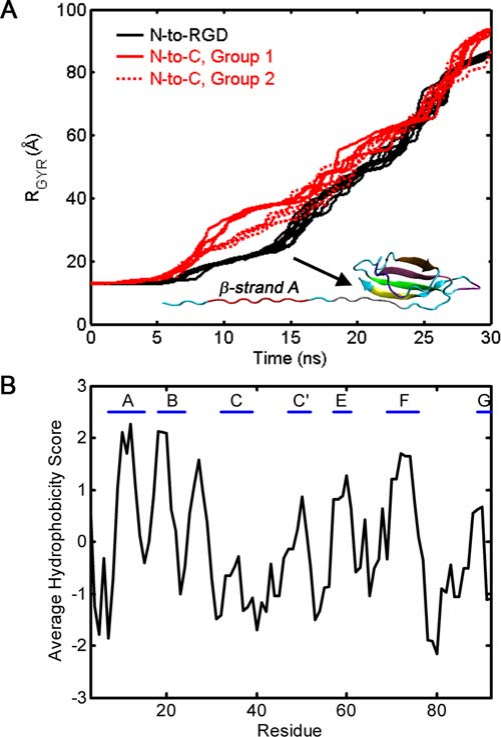
Different Unfolding Pathways for 10FNIII. (A) Backbone radius of gyration as a function of time for N-to-RGD (black) and N-to-C (red) pulling. The mass weighted radius of gyration is calculated for the backbone atoms (N, H, Cα, C, and O atoms) for all time points along the pulling trajectory. For N-to-C pulling, two groups of unfolding profiles are readily identified – red solid lines represent group 1, and red dotted lines represent group 2. Unfolding in group 1 shows larger values of extension during intermediate times. A representative structure from the pulling simulation that corresponds to the kinetic intermediate in the N-to-RGD force profile is shown (inset). The β-strands are colored as shown in Figure 1A with β-strand *A* labeled for clarity. (B) Kyte-Doolittle hydrophobicity values for β-strands in 10FNIII. With a window size of five, the Kyte-Doolittle hydrophobicity scores are computed for the 10FNIII amino acid sequence. Residue definitions for the β-strands defined in (A) are labeled above.

We explored structural variations in unfolding pathways when tensile forces are applied through the different axes. Analysis of the radius of gyration of the molecule as a function of time revealed that pulling on the RGD site leads to a single, well-defined unfolding pathway that was reliably followed in all ten unfolding simulations (Figure 3A). By contrast, when similar mechanical forces were applied through the N-to-C axis, two distinct unfolding pathways were observed (Figure 3A). Each unfolding trajectory passed through a characteristic extension plateau that corresponds to kinetic intermediates. Interestingly, despite the fact that different pulling axes are used, the unfolding profiles for both N-to-C and N-to-RGD pulling have plateaus that occur at similar times and last for similar durations (Figure 3A).

Structures taken from the time interval corresponding to the plateau in the N-to-RGD unfolding profile all have a fully solvent exposed N-terminal β-strand (one representative is shown in Figure 3A), and the exposed strands in this kinetic intermediate are the most hydrophobic β-strands in 10FNIII (Figure 3B). These observations are consistent with the notion that cell traction-mediated unfolding of 10FNIII leads to the formation of a partially unfolded intermediate that may facilitate the formation of hydrophobic contacts between different FN domains and drive fibril formation [23], [24].

**Figure 4:**
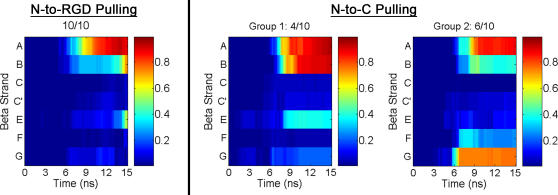
Solvent Accessibility Factor of Each β-strand During the Early Stages of Unfolding. The color scale (right), ranging between 0 and 1, represents the degree of solvent exposure of the β-strand (labeled *A* through *G*, left) for time≤15 ns. With this scaling, β-strands in the initial structures all have *f* = 0, while fully exposed strands have *f* = 1.

**Figure 5:**
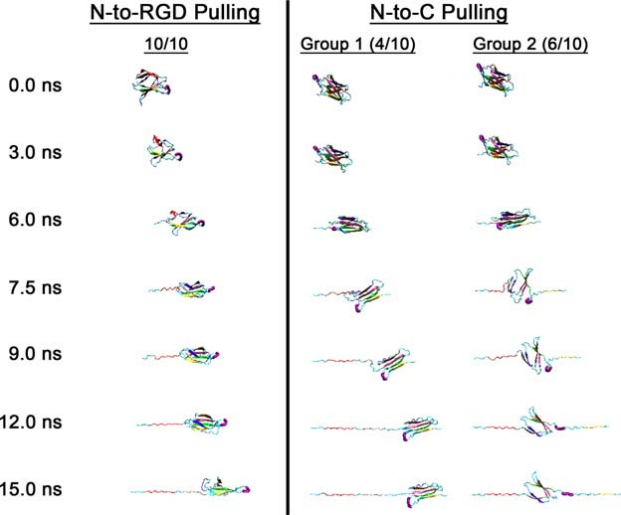
Snapshots of Early Events in the Unfolding Process (time≤15 ns). Unfolded structures show β-strand coloring corresponding to those defined in Figure 1A. The RGD loop is highlighted as a purple tube. The structures are oriented such that the N-terminal ends are on the left so that pulling of 10FNIII occurs towards the right. Unfolding as a function of time (labeled at left) is shown vertically for both N-to-RGD and N-to-C axes.

To obtain a qualitative description of the structures sampled along both axes, we analyzed the different unfolding trajectories based on the order in which each β-strand in the structure becomes solvent exposed. During N-to-C unfolding, the two types of unfolding pathways (Figure 3A) showed distinct patterns of β-strand exposure. The N-terminal β-strands became solvent exposed first in four out of ten trajectories (group 1), while in the remaining six trajectories (group 2), β-strand *G* unfolded before the N-terminal β-strand *A* (Figure 4 and Movies S1 and S2). By contrast, when tensile forces were applied along the N-to-RGD axis, β-strand *A* unfolded first in all ten trajectories (Figure 4 and Movie S3). Representative structures from the different trajectories highlight these differences (Figure 5).

### Mechanical Unfolding of 10FNIII Disrupts Integrin Binding and Induces Cell Detachment

**Figure 6:**
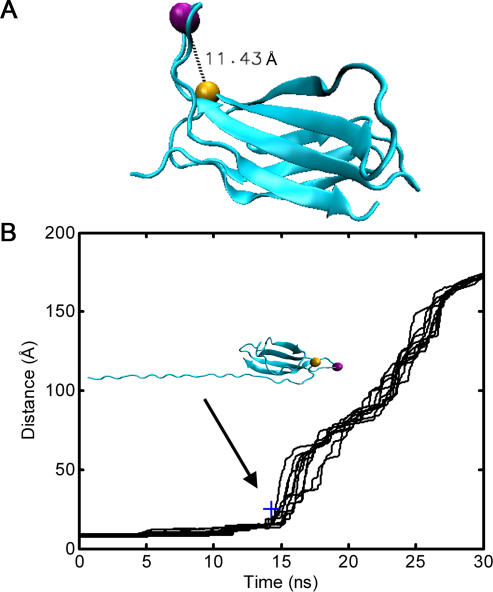
The Arginine Cell Detachment Trigger. (A) R1445 in the crystal structure of 10FNIII. Residues R1445 and G1494 (the central glycine in the RGD motif) are depicted as gold and magenta spheres, respectively. (B) Positioning of an essential arginine during the course of N-to-RGD unfolding. The distance between the Cα atoms of R1445 and G1494 is shown as a function of time for each of the ten unfolding simulations along the N-to-RGD axis. R1445 rapidly moves far from the RGD loop once the kinetic intermediate is formed (time point labeled with structure inset).

An analysis of the conformation of 10FNIII in the vicinity of the integrin-binding RGD site reveals that the protein undergoes significant conformational changes during tension-induced module unfolding. Of particular interest are specific residues near the RGD loop that have been shown to be important for high affinity binding to integrins. For example, non-conservative mutations at position R1445, which sits approximately 11 Å from the RGD loop (Figure 6A), can significantly hinder the ability of cells to bind FN fragments *in vitro* [25], [26]. R1445 therefore appears to play a major role in the formation of FN-integrin complexes, possibly by forming a secondary binding site for integrins [25], [26].

We found that early in the unfolding trajectory, the distance between R1445 and G1494 (the central glycine in the RGD motif) remained relatively constant; however, after 15 ns, this distance increased dramatically (Figure 6B). This time also corresponds to the end of the extension plateau when the kinetic intermediate forms (Figure 3A). These observations suggest that once this intermediate is generated, the affinity of FN for cell-surface integrins significantly decreases. The mechanical coupling between FN fibril formation and disruption of cell-FN adhesions may permit cells to reiterate this process of binding, pulling, and detaching on additional neighboring FN molecules. This process promotes coordinated extension of FN fibril assembly and cell spreading over time and space.

**Figure 7:**
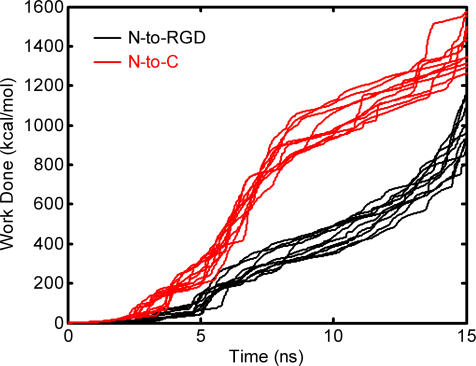
Calculation of the Work Needed to Partially Unfold 10FNIII Along Either Axis. Data for each of the 10 simulations pulled along N-to-RGD (black) and N-to-C (red) are shown for the first 15 ns.

Lastly, we note that these data suggest that 10FNIII, when subjected to mechanical unfolding at the RGD site, does not adopt structures that are more unfolded than the kinetic intermediate shown in Figure 6B. In the N-to-RGD unfolding simulations, this partially unfolded intermediate is reached around 15 ns (Figure 3A), which coincides with the mechanical decoupling of R1445 from the vicinity of the RGD loop. This structure exposes hydrophobic regions at the N-terminus that may facilitate FN fibril formation. Similarly, pulling along an N-to-C axis also results in partially unfolded kinetic intermediates that occur around 15 ns (Figure 3A), where some of these structures have solvent exposed, hydrophobic N-terminal β-strands (Figure 5). In principle, these structures can promote FN fibrillogenesis through similar hydrophobic contacts. However, a comparison of the work as a function of time required to unfold 10FNIII along the two axes reveals that the cumulative work needed to reach these kinetic intermediates is significantly less for pulling along the N-to-RGD axis (Figure 7).

## Discussion

A number of studies suggest that FN fibrillogenesis requires cell-generated traction forces that lead to the formation of partially unfolded FN intermediates [4], [8]–[11], [13], [27], [28]. Since prior experiments have provided relatively limited information regarding the precise structural changes underlying the formation of FN fibrils, we used dynamical simulations to model the early steps in the fibrillogenesis process. Unlike previous experiments that applied mechanical forces at the N- and C-termini of FN modules, we studied the effect of applying forces at the physiological site of cell binding to 10FNIII through membrane integrin receptors, and we compared our data to unfolding trajectories that were generated by pulling at the N- and C-termini. Our results demonstrate that the response of 10FNIII to mechanical strain very much depends on the axis in which tensile forces are applied and that physiological loading conditions produce a more robust response.

Earlier studies have shown that 10FNIII can unfold via several different pathways when mechanical forces are applied at the N- and C-termini, and our results are consistent with these observations [17]–[21]. In our model, pulling along an N-to-C axis yielded two distinct unfolding pathways – in one the N-terminal strand *A* was exposed first, and in the other the C-terminal strand *G* was the initial strand to become exposed. Previous atomic force microscopy experiments have identified a partially unfolded intermediate adopted during force-induced unfolding of 10FNIII that may correspond to a structure that has an exposed β-strand; either at the N-terminus (strand *A*) or the C-terminus (strand *G*) [29]. Past studies have suggested that only the pathway associated with early exposure of the *G* strand is relevant under physiological conditions based on an analysis of unfolding barrier heights [19]. By contrast, we find similar force profiles and barrier heights for both N-to-C unfolding pathways, suggesting that both pathways are equally likely to occur in 10FNIII under these loading conditions.

To gain insight into how cell traction affects the structure of 10FNIII, we modeled the interactions between the integrin receptor and the RGD motif such that force is transmitted to FN through the center Gly residue of the RGD sequence. In actuality, it is likely that the integrin-10FNIII/RGD interface involves multiple inter-atomic interactions resulting in a pulling force that may be distributed over several atoms. However, in the absence of an integrin-10FNIII crystal structure, the precise manner in which these forces are distributed over atoms in this module is unknown [30], [31]. Nevertheless, since the RGD loop is likely the principle point of contact between FN and the integrin receptor, modeling FN unfolding by pulling at a distinct atom in the RGD loop therefore represents a convenient compromise that enables unfolding simulations to be performed in an efficient manner.

In both cases of pulling along either the N-to-C or N-to-RGD axis, the work put into the system is initially used to reorient the module until the end points of the axis (the anchor point and the pulling point) are aligned along the direction of pulling (along the long axis of 10FNIII). Overall, the choice of the pulling direction for N-to-RGD pulling only changes the amount of time initially spent to reorient the molecule such that the anchor point-to-pulling point axis is aligned with the pulling direction, but the resulting unfolding pathways are thereafter similar (data not shown). Given the relative positions of the N-terminus and the RGD loop with respect to the direction of pulling, less than 3 ns are spent realigning the N-to-RGD axis at the beginning of the trajectories (compare Movies S1 and S2 to Movie S3). During this time, almost no unfolding occurs in either of the N-to-C unfolding pathways (Figure 3A, Figure 5). Therefore, the difference in the work required to unfold 10FNIII by pulling along either axis cannot be explained by mere reorientation of 10FNIII.

Partial unfolding of 10FNIII by pulling along the N-to-RGD axis requires less work than partial unfolding by puling along the N-to-C axis, at least during the early stages of the unfolding trajectory (up to the point when the kinetic intermediate is reached prior to cell detachment, time∼15 ns). In addition, N-to-RGD pulling reliably leads to partial unfolding along one well-defined trajectory. A single pathway ensures that the same kinetic intermediates are sampled when cell-derived forces are applied at the RGD site. In addition, this intermediate contains solvent exposed hydrophobic residues within β-strands *A* and *B* (Figure 3A). Additional pulling induces conformational changes in 10FNIII that promote integrin dislodgement and therefore cell detachment. Near the RGD loop is a residue, R1445, that is required for high affinity binding of FN to integrins, and its separation from the RGD loop increases following the exposure of the N-terminal strands in the kinetic intermediate [25], [26]. This conformational transition likely decreases the affinity of 10FNIII for integrins once the N-terminal strands are exposed (Figure 6B). We also note that FN contains a synergy site, located in module 9FNIII, that also facilitates FN adhesion to cell surface receptors [32]. While we do not explicitly consider the role of the module that contains the synergy site, we note that a R1445A mutation reduces the affinity of FN fragments, which contain the synergy site (7–10FNIII), to cell-surface receptors [25]. This observation is consistent with the notion that unfolding near the R1445 site facilitates cell-detachment even when the synergy site is present.

**Figure 8:**
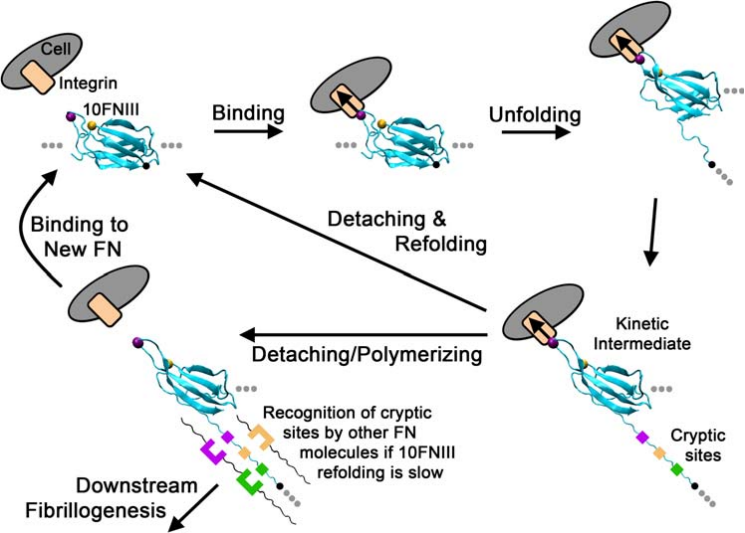
Cell-mediated Fibrillogenesis. The proposed model involves the formation of a partially unfolded intermediate during cell-traction mediated unfolding. Potential cryptic sites may exist in this partially unfolded structure that promote FN aggregation and fibrillogenesis. Subsequent unfolding past this intermediate induces a crucial arginine at a secondary binding site to move to a position far from the RGD loop. This frees the cell and enables it to reattach to another FN molecule to propagate the process. If 10FNIII refolding is relatively slow, then unfolded portions of this domain can make contacts with other FN molecules.

Here we focused on conformational changes in 10FNIII and its relationship to FN fibrillogenesis. It is worthwhile to note that other FN modules are likely involved in fibril formation. There are data to suggest that cryptic FN binding sites exist in various FNIII modules: 1FNIII, 2FNII, 7FNIII, 9FNIII, 10FNIII, and 15FNIII [13], [14], [33]–[35]. Since our unfolding simulations model 10FNIII unfolding in a dilute solution, they do not explicitly account for other interactions that are likely important for fibrillogenesis. In particular, it may be possible for other FN molecules to become incorporated into FN fibrils through interactions that involve other partially unfolded domains [13], [14], [33]–[35]. Nevertheless, our results do imply that cell traction-mediated unfolding of 10FNIII can lead to the formation of partially unfolded kinetic intermediates that can influence the rate of FN fibril formation through several different mechanisms. First, the exposure of hydrophobic residues in 10FNIII may facilitate the formation of hydrophobic contacts between distinct FN molecules, thereby promoting FN-FN association (Figure 8). Since exposure of these hydrophobic sites is quickly followed by cell-detachment, the formation of FN-FN contacts will occur only if FN refolding is slow relative to the time associated with the formation of FN-FN contacts. Secondly, our data suggest that formation of a relatively long lived kinetic intermediate may promote cell-detachment – a process which frees cell-surface integrins to bind other FN molecules. In this latter mechanism, formation of partially unfolded states that have decreased affinity for integrin receptors enables additional cycles of integrin binding to FN and subsequent incorporation of newly bound FN molecules into fibrils.

Our data also provide insight into the physical basis of the mechanical stability of 10FNIII. Additional simulations performed with electrostatic interactions turned off suggest that the main obstacle to early unfolding arises from changes in the internal geometry, rather than breaking favorable electrostatic interactions involving strand *A* (Figure S1 in Supporting Information). That is, force barriers in the unfolding profile arise mainly from short range interactions due to the internal rearrangements in the bond lengths, bond angles, and van der Waals energy (Figure S2 in Supporting Information). These conclusions are supported by recent experimental evidence that suggest that electrostatic interactions are not critical to the mechanical stability of 10FNIII because 10FNIII unfolding is not dependent on the protonation state of residues participating in hydrogen bonds [36].

Finally, it is interesting to note that our finding that the unfolding profile of 10FNIII depends on the axis in which tensile forces are applied is similar to behavior exhibited by ‘prestressed’ materials, including certain biopolymers (e.g., fibrin clots and stress fibers) and living cells under isometric tension at rest [37]–[40]. Such “prestressed” states behave quite differently depending on where and how the load is applied, and they similarly respond to applied loads by geometrically rearranging the position of their load-bearing elements [40], [41]. In particular, force spectroscopy has shown that differential protein unfolding occurs for force exerted along different axes of some proteins, as in the case of ubiquitin and E2lip3 [42], [43]. Simulations have further investigated the differential behavior of proteins stressed along different directions, as in the case of ubiquitin and titin [44], [45]. Protein models that are based on this formalism can be used to understand and predict the unfolding behavior of proteins subjected to external forces, and variations in the internal stability of the protein can be deduced from force-induced unfolding trajectories of the molecule [40], [41], [46]. Unfolding along a single well-defined pathway that contains a partially unfolded state having solvent N-terminal strands suggests that significant internal strain is present along the N-to-RGD axis – strain that leads to partial unfolding in response to an external force preferentially oriented along this axis. The modeling approach used here may therefore prove useful for analysis of the molecular biophysical basis of cellular mechanotransduction as well as ECM remodeling.

## Materials and Methods

### The Initial Model

The structure of 10FNIII (residues Val1416 to Thr1509) was taken from the crystal structure of 7–10FNIII (PDB ID 1fnf) [30], [47]–[49]. An initial polar-hydrogen model of 10FNIII was made with CHARMM [50]. The N-terminus of the protein was acetylated and the C-terminus was amidated with a methyl amine. Atoms in the N and C-terminal blocked regions were energy minimized, while keeping the remainder of the protein fixed, for 100 steps of steepest descent minimization. The full protein was then minimized for 200 steps of steepest descent minimization followed by 10 steps of Adopted Basis Newton-Raphson. We refer to this energy minimized structure as the reference structure. The reference structure has a mass-weighted backbone (N-Cα-C) root-mean-square (RMS) deviation of 0.2 Å from the unminimized crystallographic structure. All calculations used the implicit solvent model EEF1 [51]–[53].

### Generating Representative Structures

To generate different starting structures for the pulling simulations, we performed a relatively short MD simulation on the isolated 10FNIII structure. A time step of 0.001 ps was used, and the equations of motion were integrated with a leap frog integrator. The temperature of the system was maintained by coupling the system to a heat bath at 300 K using the Berendsen method [54]. Equilibration consisted of 0.2 ns of simulation time followed by 0.9 ns of production dynamics. Ten equally spaced structures (100 ps apart) were extracted from the production dynamics, and these 10 structures were the initial structures for the pulling experiments. These conformations had an average backbone-atom mass-weighted RMS deviation of (2.8±0.1)Å from the reference structure.

### Constant Velocity Steered Molecular Dynamics

Constant velocity SMD simulations were performed by placing the Cα atom of the pulling point at the origin and the dummy atom on the z-axis. In this framework, pulling along an N-to-RGD axis corresponds to pulling the dummy atom along the +z direction. For N-to-C pulling, the protein is oriented such that the C-terminus is the pulling point and the N-terminus is the fixed point. In this framework, N-to-C pulling corresponds to pulling the C-terminus in the −z direction.

In constant velocity SMD external force at the pulling point is provided by translating, at constant velocity, the free end of a spring (dummy atom in Figure 1C,D) that is attached to the pulling point. The rate at which 10FNIII unfolds is therefore a function of the velocity of the dummy atom. A pulling speed of v = 0.01 Å/ps for both N-to-RGD and N-to-C pulling was used. In all pulling simulations the Cα atom of the N-terminus (Val1416) is fixed, and the pulling vector is defined by the positions of the pulling point and dummy atom (i.e. along the z-axis). The endpoints of the spring correspond to the pulling point (the Cα atom of either Gly1494 at the RGD loop or Thr1509 at the C-terminus for N-to-RGD or N-to-C pulling, respectively) and the dummy atom. A spring constant of k = 0.6 kcal/mol/Å^2^ was used. Dynamics were run as described above for the initial structure generation except that simulations were run for a total of 30 ns. The system temperature was maintained by coupling to a heat bath at 300 K, again using the Berendsen method [54]. During the pulling simulations, the temperature of the system remained near this target value as instantaneous temperatures were within 5% of 300 K during the last 29.8 ns of the simulation. Coordinates were saved every 0.2 ps, and SHAKE was used to hold hydrogen bond lengths near their equilibrium values [55].

Constant-velocity SMD pulling simulations were performed on each of the ten initial structures along both an N-to-RGD and N-to-C axis. Each simulation was run for 30 ns as outlined above, making the total simulation time, for the 20 structures, 600 ns. Unfolding trajectories and associated structures were visualized with the software program VMD (version 1.8.5) [56]. Some simulations were performed using a potential energy function where specific electrostatic interactions were set to zero (Text S1). This was achieved by setting the charges for the atoms from Arg1421 to Thr1429 (i.e., β-strand *A*) to zero. One N-to-RGD pulling simulation was performed under this altered energy function.

### Force Calculations

The NOE facility in CHARMM was used to introduce an external pulling force on the dummy atom of interest, where KMIN = 0 kcal/mol/Å^2^, KMAX = 0.6 kcal/mol/Å^2^, RMIN = 0 Å, RMAX = 0 Å, FMAX = 3000 kcal/mol/Å [50]. The result is to add an additional term to the potential energy function: 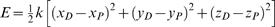 where (*x_D_*,*y_D_*,*z_D_*) and (*x_P_*,*y_P_*,*z_P_*) are the Cartesian coordinates of the dummy and pulling atoms, respectively. The components of the force on the pulling atom can be expressed as:
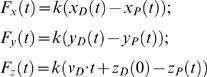
(1)where k is the spring constant. We note that the last expression in Equation 1 follows from the fact that the dummy atom is pulled at a constant velocity, *v_D_*, along the z-axis, so that *z_D_*(*t*) = *v_D_*·*t*+*z_D_*(0). With these definitions the magnitude of the total force on the pulling atom is given by 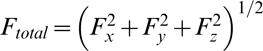.

The terms in the potential energy function governing the unfolding simulation give rise to the internal forces that resist the pulling. More concretely, the force associated with the total internal energy, which is the sum of the individual energy contributions attributed to the bonded (bond, angle, dihedral angle, and improper planar deformation energies) and non-bonded (van der Waals, electrostatic, and EEF1 solvation energy) interactions, can be calculated for all the atoms in the protein throughout the unfolding trajectory. The magnitude of the internal force of 10FNIII that contribute to its mechanical stability is then calculated as described above.

### Calculating the Work Done

We define the work needed to separate the anchor point and the pulling point by a distance *R*, *W(R)*, to be:
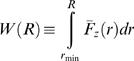
(2)where *r_min_* is the value of the distance between the anchor point and the pulling point at t = 0. The function *F̅* *_z_*(*r*) is given by:
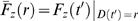
(3)where *D*(*t*′) is the distance between the anchor point and the pulling point at time *t*′. Values of *W(R)* are computed from Equation 2 using the trapezoidal rule.

### Calculating the Solvent Accessibility Factor

Solvent accessible surfaces of the β-strands in the structures from the unfolding trajectories were computed using a Lee and Richard's algorithm with a 1.4 Å sphere radius and 0.025 Å accuracy [57]. β-strand boundaries are as defined in [17]. For each time point, we compute a solvent accessibility factor, *f*, as follows:
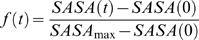
(4)where *SASA(0)* and *SASA(t)* are the solvent accessible surface areas (SASAs) of the given β-strand at times *0* and *t*, respectively. *SASA*_max_ is the β-strand's maximum solvent accessible surface area and is computed by calculating the SASA of the strand in the initial structure when all other atoms in the protein are removed. With this definition, β-strands in the initial structures all have *f* = 0, while fully exposed strands have *f* = 1.

## Supporting Information

Text S1 (0.04 MB DOC)

Figure S1 Evaluating the Role of Electrostatic Interactions Involving β-strand A. (A) Snapshots of the N-to-RGD unfolding trajectory with (right) and without (left) strand A electrostatic contributions ignored. Comparison of the unfolding trajectories for the same initial conditions, but calculated for either the normal energy potential (U) or the modified electrostatic energy potential (UE), show minimal differences between the unfolded structures. (B) Solvent accessibility factor of the β-strands. The pattern of solvent accessibility of the secondary structural elements (labeled at left) calculated every 3 ns throughout the trajectory show similarities between the two N-to-RGD unfolding profiles for normal and modified electrostatics.
(0.92 MB DOC)

Figure S2 Energy Contributions Significant for 10FNIII Mechanical Stability Along the N-to-RGD Axis. (A) Electrostatic energy contributions to the force profile. The black curve denotes the N-to-RGD force profile with the full potential energy function. For the same starting structure, the force profile (magenta) is calculated with the electrostatic interactions involving strand A turned off as outlined in the text. (B) Contributions due to bond length, bond angle, and van der Waals energy terms to the force profile. The black curve represents the magnitude of the external pulling force needed to unfold 10FNIII. The blue curve is the magnitude of the sum of the bond length, bond angle, and van der Waals energy contributions to the calculated internal force for the same initial structure.
(0.66 MB DOC)

Movie S1 Partial N-to-C Unfolding, Group 1. The anchor and pulling points are highlighted as black spheres with pulling towards the right. Initial unfolding of 10FNIII occurs at the N-terminus.
(6.10 MB MOV)

Movie S2 Partial N-to-C Unfolding, Group 2. The anchor and pulling points are highlighted as black spheres with pulling towards the right. Initial unfolding of 10FNIII occurs at both the N- and C- termini.
(5.87 MB MOV)

Movie S3 Partial N-to-RGD Unfolding. The anchor and pulling points are highlighted as black spheres with pulling towards the right. Initial unfolding of 10FNIII occurs exclusively at the N-terminus. The central glycine is labeled with a magenta sphere while Arg1445 is highlighted with a gold sphere. Note that the separation of these two residues increases following the occurrence of the kinetic intermediate (paused frame).
(7.34 MB MOV)
