# Supplementary material for: Fibronectin Unfolding Revisited: Modeling Cell Traction-Mediated Unfolding of the Tenth Type-III Repeat
Source: PLoS One. 2008 Jun 11;3(6):e2373. doi: 10.1371/journal.pone.0002373 (PMC2585069; doi:10.1371/journal.pone.0002373)
Supplement: Text S1 — (0.04 MB DOC) [file pone.0002373.s001.doc]

**Supporting Information**

***Electrostatic Interactions and Unfolding Along the N-to-RGD Axis***

To form the partially-unfolded intermediate shown in Figure 3A, β-strand *A* must first detach from its corresponding β-sheet. Since strands within this β-sheet share hydrogen-bonds as well as a number of van der Waals contacts, we explored the relative contribution of different inter-atomic interactions to the overall force profile.

In the solvent model used for these simulations, hydrogen-bonds are primarily modeled through electrostatic interactions between donor and acceptor dipoles [1]. To investigate the relative importance of these electrostatic interactions, we conducted additional unfolding simulations where the electrostatic contributions between strand *A* and the remainder of the protein (as well as between atoms within strand *A*) were explicitly set to zero. For these trajectories, the expression for the electrostatic interaction energy *U_E_* between atoms *i* and *j* is

(1) $U_{E}(q_{i},q_{j})=\left\{\begin{matrix} \frac{q_{i}q_{j}}{\varepsilon_{D}r_{ij}} i \text{and} j\notin\text{strand} A \\ 0 \text{otherwise} \end{matrix}\right.$

where *q_i_* and *q_j_* denote the charges on atoms *i* and *j*, respectively, *r_ij_* is the distance between atoms *i* and *j*, and ε_D_ is the dielectric constant (in the EEF1 implicit solvent model *ε_D_ = r_ij_* [1-3]). Equation 1 ignores electrostatic energy contribution between atoms in strand *A* and between atom pairs with one member in strand A and the other not.

Mechanical unfolding simulations with Equation 1 yield an unfolding pathway that is nearly identical to that obtained with the full potential energy function (Figure S1). Moreover, when the electrostatic contribution is ignored, the magnitude and shape of the time-dependent force profile is comparable to simulations in which all electrostatic interactions are included (Figure S2A). These findings imply that electrostatic contributions involving strand *A* do not play a significant role in the overall 10FNIII unfolding force profile. In addition, they suggest that the main obstacle to early unfolding arises from changes in the internal geometry, rather than breaking favorable electrostatic interactions involving strand *A*. An analysis of the different contributions to the force profile obtained with the full potential are consistent with these observations and suggest that force barriers in the unfolding profile arise mainly from short range interactions due to the internal rearrangements in the bond lengths, bond angles, and van der Waals repulsion (Figure S2B).

**References**

**1. Lazaridis T, Karplus M (1999) Effective energy function for proteins in solution. Proteins 35: 133-152.**

**2. Lazaridis T, Karplus M (1999) Discrimination of the native from misfolded protein models with an energy function including implicit solvation. J Mol Biol 288: 477-487.**

**3. Lazaridis T, Karplus M (1997) "New view" of protein folding reconciled with the old through multiple unfolding simulations. Science 278: 1928-1931.**

**Funding Sources**

This work was supported by the NIH (NIH/NIGMS Molecular Biophysics Training Grant T32 GM008313 and NIH CA45548) as well as by the Keck Foundation (NAKFI Nano 06).
