# Supplementary figures and images for: Fibronectin Unfolding Revisited: Modeling Cell Traction-Mediated Unfolding of the Tenth Type-III Repeat
Source: PLoS One. 2008 Jun 11;3(6):e2373. doi: 10.1371/journal.pone.0002373 (PMC2585069; doi:10.1371/journal.pone.0002373)

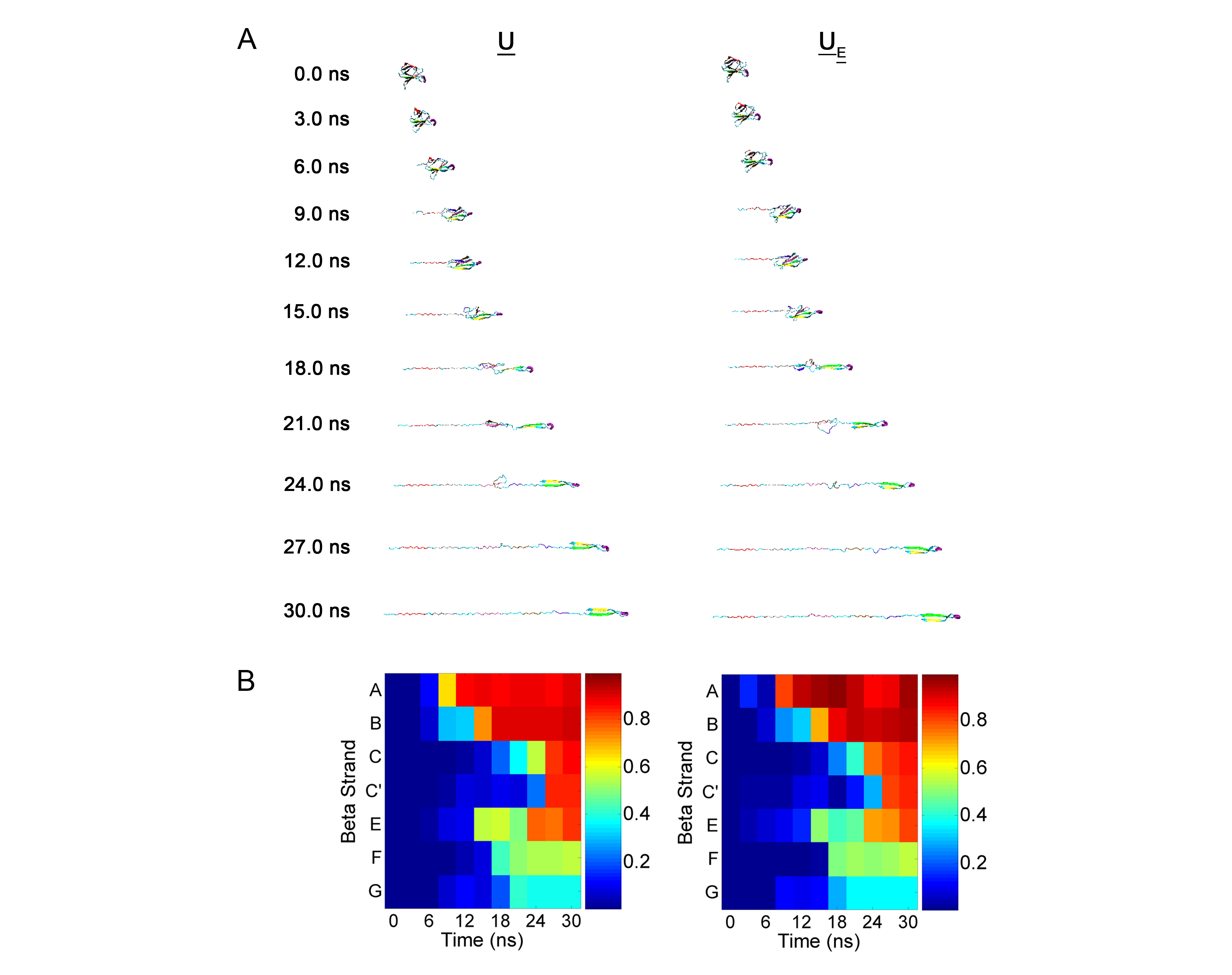

Supplement: Figure S1 — Evaluating the Role of Electrostatic Interactions Involving β-strand A. (A) Snapshots of the N-to-RGD unfolding trajectory with (right) and without (left) strand A electrostatic contributions ignored. Comparison of the unfolding trajectories for the same initial conditions, but calculated for either the normal energy potential (U) or the modified electrostatic energy potential (UE), show minimal differences between the unfolded structures. (B) Solvent accessibility factor of the β-strands. The pattern of solvent accessibility of the secondary structural elements (labeled at left) calculated every 3 ns throughout the trajectory show similarities between the two N-to-RGD unfolding profiles for normal and modified electrostatics. (0.92 MB DOC) [file pone.0002373.s002.tif]

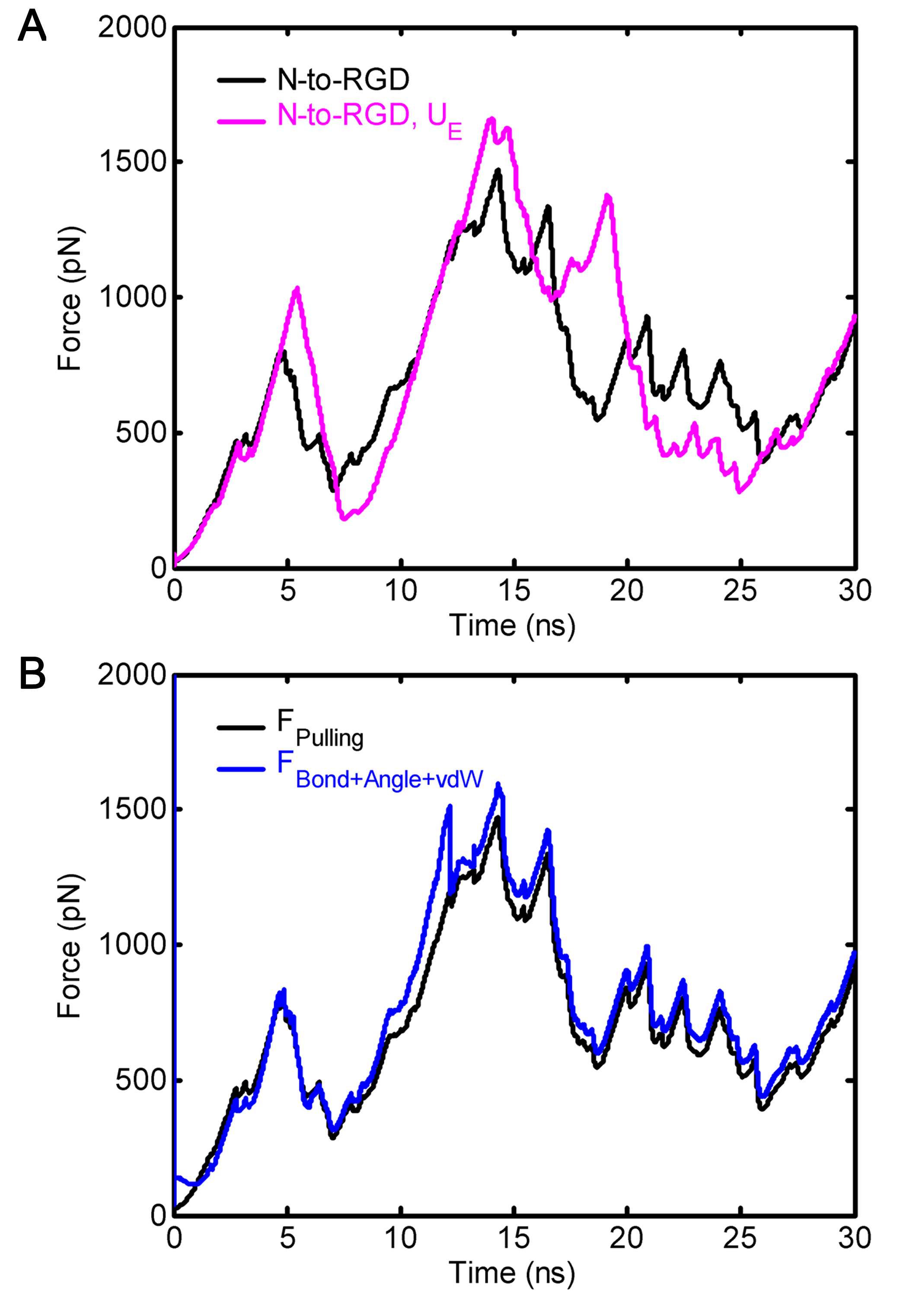

Supplement: Figure S2 — Energy Contributions Significant for 10FNIII Mechanical Stability Along the N-to-RGD Axis. (A) Electrostatic energy contributions to the force profile. The black curve denotes the N-to-RGD force profile with the full potential energy function. For the same starting structure, the force profile (magenta) is calculated with the electrostatic interactions involving strand A turned off as outlined in the text. (B) Contributions due to bond length, bond angle, and van der Waals energy terms to the force profile. The black curve represents the magnitude of the external pulling force needed to unfold 10FNIII. The blue curve is the magnitude of the sum of the bond length, bond angle, and van der Waals energy contributions to the calculated internal force for the same initial structure. (0.66 MB DOC) [file pone.0002373.s003.tif]
